# Early Post-Transplant Peripheral B-Cell Profiles in Kidney Transplant Recipients: Clinical Associations and Limitations

**DOI:** 10.3390/jcm15114064

**Published:** 2026-05-24

**Authors:** Ariadni Fouza, Maria Daoudaki, Anneta Tagkouta, Persefoni Talimtzi, Georgios Tsoulfas, Nikolaos Antoniadis, Asimina Fylaktou

**Affiliations:** 1 Department of Transplant Surgery, Center for Research and Innovation in Solid Organ Transplantation, School of Medicine, Faculty of Health Sciences, Aristotle University of Thessaloniki, 54124 Thessaloniki, Greece; ariadnefou@gmail.com (A.F.); tsoulfasg@auth.gr (G.T.); nikanton@auth.gr (N.A.); 2 Laboratory of Biological Chemistry, School of Medicine, Faculty of Health Sciences, Aristotle University of Thessaloniki, 54124 Thessaloniki, Greece; taganneta@hotmail.com; 3 Department of Hygiene, Social-Preventive Medicine and Medical Statistics, School of Medicine, Faculty of Health Sciences, Aristotle University of Thessaloniki, 54124 Thessaloniki, Greece; 4 Department of Immunology, National Peripheral Histocompatibility Center, Hippokration General Hospital of Thessaloniki, 54642 Thessaloniki, Greece; fylaktoumina@gmail.com

**Keywords:** B cell subpopulations, class switch memory B cells, kidney transplant rejection, naïve to memory B-cell ratio, immune monitoring, clustering

## Abstract

**Background:** The clinical relevance of circulating B-cell subpopulations during the early period after kidney transplantation remains incompletely understood. **Methods:** In this prospective single-center study, frequencies and absolute numbers of peripheral B-cell subpopulations were longitudinally assessed by flow cytometry in 71 kidney transplant recipients before transplantation (T0) and at 3 (T3), 6 (T6) and 12 months (T12) post-transplant. Associations with graft function, rejection episodes and clinical variables were explored. **Results:** During the first post-transplant year, relative frequencies of total and naïve B cells declined, whereas absolute counts showed modest increases. Memory B-cells expanded over time, driven by both class-switched (CSBC) and class-non-switched (CNSBC) subsets. Transitional regulatory B cells (tBregs) and plasmablasts decreased significantly, while memory regulatory B cells (mBregs) remained stable. Pre-transplant B-cell profiles did not differ between recipients experienced rejection and those with stable graft function. At T12, rejection was associated with a shift toward a memory-dominant peripheral profile, characterized by reduced naïve representation. tBregs showed modest positive associations with graft function during follow-up. Hierarchical clustering identified naïve- and memory-dominant phenotypes representing distinct post-transplant immune compositions. **Conclusions:** Early post-transplant peripheral B-cell landscapes are dynamic and heterogeneous. Peripheral B-cell phenotyping shows limited value as a standalone clinical monitoring tool.

## 1. Introduction

Survival and quality of life are markedly improved in patients with end-stage renal disease following kidney transplantation. However, graft dysfunction and loss are frequently driven by immune-mediated rejection and related alloimmune processes. Despite advances in immunosuppressive therapy, early rejection still occurs, reflecting the complexity and heterogeneity of alloimmune responses. These challenges have sustained interest in immune monitoring strategies aimed at better characterizing post-transplant immune status, including the study of circulating B-cell subpopulations [1,2]. Although numerous studies have examined how B-lymphocyte populations evolve following kidney transplantation, the clinical relevance of these changes and their temporal relationship with rejection and graft function remain unclear [3,4,5].

Circulating B-cell subpopulations are increasingly recognized as markers of immune reconstitution and activation, with much of the existing literature focusing on their role in antibody-mediated rejection. B-cells can also exert antibody-independent effects through antigen presentation, cytokine secretion and co-stimulatory signaling, thereby shaping T-cell responses, immune regulation and tolerance [6,7,8]. These diverse functions, together with the dynamic nature of post-transplant immune adaptation, make it challenging to link peripheral B-cell phenotypes to specific clinical outcomes.

B-cells are a heterogeneous population distinguished by developmental stage, surface phenotype and functional specialization [1,6,9]. Peripheral maturation generates transitional, naïve, memory and antibody-secreting subsets. Transitional B cells as recent bone marrow emigrants, differentiate into mature naïve B cells (IgD+CD27−) [1,10,11], which on antigen exposure give rise to memory B cells, plasmablasts (CD27++CD38++IgD+/−) and plasma cells [10,12,13]. Circulating memory B cells are further classified into CNSBC (IgD+CD27+), CSBC (IgD−CD27+) and double-negative (IgD−CD27−) populations [10,14].

Beyond conventional subsets, regulatory B cells (Bregs) are a functionally defined, heterogeneous population arising at multiple stages of B-cell maturation, with classification limited by the absence of a unique surface marker or lineage-specific transcription factor [11,14]. Functionally, Bregs promote immune tolerance by enhancing regulatory T-cell activity and suppressing pro-inflammatory Th1, Th2 and Th17 responses [8,15,16], primarily through production of IL-10, TGF-β and IL-35 [8,10,17]. Among human Bregs, two subsets have been most extensively characterized: tBregs (CD19+CD24++CD38++) first described by Blair et al. [18] and mBregs with (CD19+CD24hiCD27+), functionally comparable to murine IL-10-producing B10 cells [19]. Importantly, phenotypic identification alone does not reliably indicate regulatory capacity and functional assessment often requires ex vivo stimulation to detect cytokine-producing cells [20].

In the context of transplantation, both tBregs and mBregs are of interest. In particular, tBregs have been associated with immune regulation and long-term graft survival in certain patient populations [8,15,16], although their role early post-transplant under contemporary immunosuppression remains uncertain.

In this study, we prospectively examined circulating B-cell subpopulations using flow cytometry during the first year after kidney transplantation. Our aims were to characterize early longitudinal changes in peripheral B-cell immune landscapes, explore their clinical associations, and identify the heterogeneity and limitations inherent to peripheral B-cell monitoring in routine transplant practice. To this end, we integrated longitudinal immune profiling with clinical parameters, graft function and rejection episodes.

## 2. Methods

### 2.1. Study Population

This prospective, single-center cohort study included 71 adult kidney transplant recipients (51 men and 20 women, aged 18–60 years). No preformed donor-specific anti-HLA antibodies (DSA) were detected in any recipient prior to transplantation. Pre-transplant immunological evaluation included screening for anti-HLA antibodies using standard solid-phase assays. Only recipients without detectable DSAs and with a negative complement-dependent cytotoxicity crossmatch were eligible for transplantation. All recipients underwent primary kidney transplantation and with all transplants performed under ABO-compatible conditions and with a negative crossmatch.

Patients with a history of malignancy, more than one kidney transplantation, autoimmune or hematologic disease, treatment with anti-B or anti-T lymphocyte monoclonal antibodies within 3 years, or active infection in the 3 months before transplantation were excluded. Demographic, clinical and transplant-related data were collected longitudinally before and after transplantation.

A total of 90 recipients were initially enrolled. Following withdrawals and exclusions due to missing records, relapse of the primary disease, or infection during the follow-up period, the final study cohort consisted of 71 recipients with complete data.

### 2.2. Study Design and Clinical Data Collection

Peripheral blood samples were obtained at baseline (T0, pre-transplant) and at 3, 6, and 12 months post-transplant (T3, T6, T12) for flow cytometric analysis of B-cell subpopulations including naïve, memory, regulatory, plasmablasts. Longitudinal changes in B cell frequencies and absolute numbers were assessed. Renal allograft function was evaluated at T3, T6, and T12 using estimated glomerular filtration rate (eGFR) calculated with CKD-EPI 2021 equation. Clinical variables recorded during follow up included rejection episodes, delayed graft function (DGF), infectious complications, immunosuppressive regimens and modifications, adverse events, and patient survival. Cold ischemia time (CIT) was also recorded.

The diagnosis of rejection episodes was made on the basis of biopsy findings interpreted in conjunction with clinical presentation and response to anti-rejection therapy, reflecting routine clinical practice. Due to the frequent occurrence of mixed or indeterminate histological patterns and variability in biopsy quality, formal Banff grading and strict subclassification of rejection phenotypes were not uniformly available. When possible, rejection episodes were categorized based on the available clinical and histological descriptions.

#### Immunosupressive Therapy

Immunosuppressive therapy followed the institutional protocol including risk-adapted induction and standard triple maintenance therapy. Induction therapy consisted of an anti-CD25 antibody (basiliximab), administered to 63 recipients at 20 mg pre-transplant and on postoperative day 4 together with perioperative methylprednisolone (500 mg) in standard-risk patients, whereas 8 recipients with increased immunological risk received anti-thymocyte globulin (ATG, 1.5 mg/kg/day for 4 days; cumulative dose ≈ 6 mg/kg).

Maintenance immunosuppression consisted of tacrolimus (target trough 6–8 ng/mL during the first year), mycophenolate mofetil (2 g/day initially, reduced to 1 g/day), and corticosteroids administered as intravenous methylprednisolone followed by oral tapering until day 42. Rejection episodes were treated with anti-thymocyte globulin, (ATG) for T cell-mediated rejection and corticosteroids, plasma exchange, and ± intravenous immunoglobulin for antibody-mediated rejection. Corticosteroids were gradually tapered during follow-up, and no systematic withdrawal or major modification of maintenance immunosuppression occurred except in the context of rejection treatment.

### 2.3. Ethics

The protocol was approved by the Institutional Review Board of the Medical School, Aristotle University of Thessaloniki (protocol 4356, 26 January 2021) and conducted in accordance with the Declaration of Helsinki. Written informed consent was obtained from all participants.

### 2.4. Flow Cytometry

Whole blood was stained with fluorochrome-conjugated monoclonal antibodies and analyzed on an 8-color Navios cytometer (Beckman Coulter, Marseille, France). Circulating B cell subpopulations were identified based on established surface marker expression and standardized gating strategies, as previously described [21,22]. Detailed antibody panels, fluorochrome combinations, and gating procedures are provided in Supplementary Data, Supplementary Table S1 and Figure S1.

### 2.5. Cluster Analysis

To explore peripheral immune phenotypes, transplant recipients were considered as the observational units and the frequencies of the B-cell subpopulations at one year post-transplantation were standardized using the Z-score method and analyzed using agglomerative hierarchical clustering (Euclidean distance and Ward’s method), as previously reported [23]. The optimal cluster combination was defined by maximizing the average silhouette score. All clustering analyses were conducted in R (version 4.3).

### 2.6. Statistics

Continuous variables are presented as mean ± standard deviation (SD) or median with interquartile range (IQR), as appropriate, and categorical variables as number (percentage). Comparisons between two independent groups were performed using the Mann–Whitney U test (Wilcoxon rank-sum test) for continuous variables and the chi-square or Fisher’s exact tests for categorical variables, as appropriate. Longitudinal changes in B cell subpopulations were analyzed with Friedman’s analysis of variance followed by post hoc pairwise comparisons.

For analyses following hierarchical clustering, cluster membership was treated as a categorical grouping variable. Renal function (eGFR) and other continuous clinical and immunological variables were compared between clusters using the Mann–Whitney U test (Wilcoxon rank-sum test).

Associations between B-cell subpopulations and clinical variables including recipient age, donor type, CIT, DGF and dialysis vintage, were examined using univariate and multivariate linear regression models.

Receiver operating characteristic (ROC) curve analysis was used to explore the discriminative performance of selected B-cell subpopulations in relation to rejection episodes. Correlations between B-cell subset frequencies and eGFR were assessed using Spearman’s rank correlation coefficient, and *p*-values were adjusted for multiple comparisons to control for type I error.

To evaluate the potential effect of recipient age on baseline immune profiles, B-cell subpopulations at T0 were additionally analyzed according to age strata defined by the cohort median age. A two-sided *p*-value ≤ 0.05 was considered statistically significant. All statistical analyses were conducted in R (version 4.3).

### 2.7. Declaration of AI

Artificial intelligence tools were used to help format the references according to the journal’s guidelines, as well as to prepare and improve the figures.

## 3. Results

### 3.1. Characteristics of the Study Population

Seventy-one kidney transplant recipients were included in the study, of whom 50 (70%) received grafts from deceased donors and 21 (30%) from living donors. Seven patients (9.9%) underwent preemptive transplantation without prior dialysis. Among recipients with previous renal replacement therapy, hemodialysis was more frequent than peritoneal dialysis (80.9% vs. 19.1%), with a median dialysis duration of 87 months (IQR 34–127).

All participants underwent peripheral B-cell immunophenotyping at baseline (T0) and were prospectively followed at 3 (T3), 6 (T6), and 12 months (T12) after transplantation. Maintenance immunosuppression consisted of corticosteroids, tacrolimus, and mycophenolate mofetil in all patients. The induction therapy administered to the 63 recipients (88.7%) included basiliximab while the remaining 8 recipients (11.3%) received antithymocyte globulin (ATG).

Delayed graft function occurred in 21 patients (29.6%). During the first post-transplant year, 11 recipients (15.5%) experienced rejection episodes. The baseline demographic, clinical, and transplant-related characteristics are summarized in Table 1.

### 3.2. Longitudinal Kinetics of B-Cell Subpopulations

Circulating B-cell subpopulations were assessed by flow cytometry at T0, T3, T6, and T12. Both frequencies (%) and absolute numbers (cells/µL) were analyzed longitudinally and compared with baseline values (Table 2 and Table S2).

**Figure 1:**
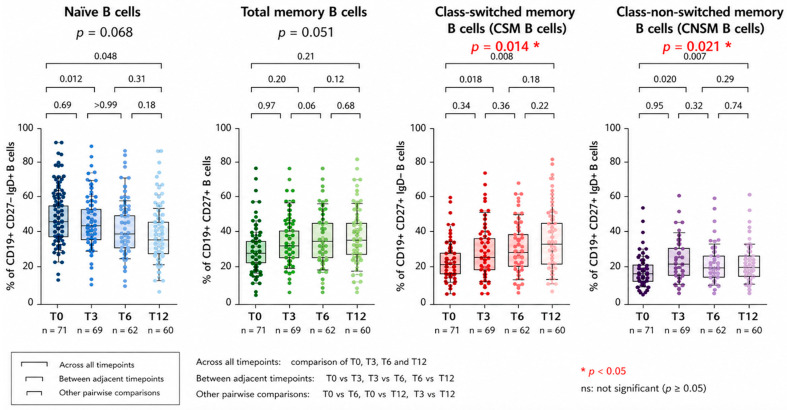
Longitudinal changes in naïve and memory-related B-cell subsets during the first year post-transplant. Dot-and-box plots showing the distribution of selected B-cell subpopulations including naïve B cells, total memory B cells, class-switched memory B cells, and class-non-switched memory B cells at T0, T3, T6, and T12. Each dot represents one patient. Overall longitudinal comparisons were performed using Friedman’s test. Exact *p*-values are displayed within each panel. Significant comparisons are indicated by * *p* < 0.05.

Over the first post-transplant year, the relative frequency of total circulating B lymphocytes declined significantly (*p* < 0.001), whereas absolute B-cell numbers increased modestly over time (*p* = 0.04). Memory B cells exhibited a significant increase in both frequency and absolute number (*p* = 0.05 and *p* = 0.03, respectively), driven by expansion of both class-switched memory (CSM, *p* = 0.01) and class non-switched memory (CNSM, *p* = 0.02) subpopulations, Figure 1. In contrast, naïve B cells demonstrated a non-significant decline in frequency, accompanied by a trend toward increased absolute counts.

Regulatory B-cell subsets displayed divergent longitudinal patterns. tBregs declined markedly in both frequency and absolute number at all post-transplant time points compared with baseline (*p* < 0.001). In contrast, mBregs remained relatively stable throughout follow-up. Plasmablasts showed a significant reduction in both frequency (*p* = 0.02) and absolute numbers (*p* = 0.03), particularly evident by 12 months post-transplant, (Table 2 and Table S2).

Taken together, these findings indicate that changes in relative frequencies did not uniformly parallel changes in absolute cell numbers. One year after transplantation, the peripheral B-cell compartment was characterized by an overall reduction in relative B-cell frequency, alongside an expansion of memory B-cell subpopulations, and a contraction of tBregs and plasmablasts.

Dot-and-box plots showing the distribution of selective B-cell subpopulations including naïve B cells, total memory B cells, class-switched memory B-cells, and class-non-switched memory B cells at T0, T3, T6, and T12. Each dot represents one patient. Overall longitudinal differences were assessed using Friedman’s test. (naïve: *p* = 0.068; total memory: *p* = 0.051; class-switched memory: *p* = 0.014; class-non-switched memory: *p* = 0.021).

A sensitivity analysis excluding recipients who received ATG induction therapy (*n* = 8) showed similar longitudinal patterns of B-cell subsets (Supplementary Table S3). The main findings—including temporal changes in total B cells, naïve B cells, total and switched memory B cells, plasmablasts, and tBregs remained significant, indicating that ATG exposure did not materially influence the principal results.

Longitudinal evaluation demonstrated temporal changes in B-cell composition during the first year after transplantation. Recipients who experienced rejection tended to exhibit lower frequencies of naïve B cells and relatively higher proportions of memory B-cell subsets over time, particularly switched memory B cells. In contrast, patients with stable graft function maintained higher levels of naïve B cells during follow-up. Transitional regulatory B cells showed greater variability but tended to be reduced in the rejection group at later timepoints, Table S4, Figure S2.

### 3.3. B-Cell Subpopulations and Renal Function over 12 Months

Associations between circulating B-cell subpopulations (frequencies and absolute counts) and renal allograft function, assessed by estimated glomerular filtration rate (eGFR), were examined at 3, 6, and 12 months post-transplantation (T3, T6, and T12).

No significant associations were observed between B-cell subsets and eGFR at T3.

At T6, two B-cell subpopulations demonstrated statistically significant associations with renal function (Table 3). tBregs were positively associated with eGFR, both in terms of relative frequency (r = 0.27, *p* < 0.001) and absolute numbers (r = 0.31, *p* < 0.001). In contrast, plasmablasts were negatively associated with eGFR (frequency: r = −0.17, *p* = 0.012; absolute numbers: r = −0.29, *p* = 0.009).

At T12, tBreg frequency remained positively associated with eGFR (r = 0.653, *p* = 0.04). The association between absolute tBreg numbers and eGFR at this time point was weak and did not reach statistical significance. No other B-cell subpopulations showed consistent associations with graft function at T12, Table 3.

Overall, among the B-cell subsets examined, tBregs were the only population to demonstrate reproducible associations with renal function during follow-up. However, these associations were modest and variable over time, underscoring the complexity of linking peripheral B-cell phenotypes to graft function in the early post-transplant period.

### 3.4. Influence of Clinical and Transplant Variables on B-Cell Subpopulations

We evaluated the association of recipient-, donor-, and transplant-related factors with B-cell subpopulation distributions at T3, T6, and T12, including recipient age, donor type (living vs. deceased), dialysis vintage, cold ischemia time (CIT), and delayed graft function (DGF). Univariate models were performed, and variables with *p* < 0.20 were entered into multivariate analyses (Supplementary Tables S5 and S6).

Because aging is known to influence the balance between naïve and memory B-cell compartments, baseline B-cell subsets were additionally evaluated according to recipient age. Stratification based on the cohort median age did not reveal significant differences in total B cells, naïve B cells, or memory B-cell subsets at baseline. However, older recipients exhibited slightly higher frequencies of mBregs.

Detailed results are presented in Supplementary Data, Table S7.

At T12, univariate analyses suggested several associations. Recipient age was associated with the frequency of total B cells and with absolute numbers of total B cells, naïve B cells, total memory B cells (including CSM and CNSM), and mBregs. Dialysis vintage correlated with absolute numbers of total B cells, naïve B cells, and plasmablasts. CIT was associated with absolute numbers of total memory and CNSM B cells, while donor type and DGF were associated with absolute numbers of memory B-cell subsets, including CSM and CNSM.

However, none of these associations remained statistically significant in multivariate models, indicating that longitudinal B-cell kinetics were not independently explained by the evaluated clinical variables within this cohort.

### 3.5. B-Cell Subpopulations and Rejection Episodes

During the one-year follow-up, 11 recipients (15.5%) experienced biopsy-proven rejection (Group 1), whereas 60 recipients (84.5%) maintained stable graft function (Group 2), Table S8. Baseline characteristics were similar between groups (Table S8), although delayed graft function occurred more frequently in Group 1 (*p* = 0.072).

Based on the available clinical and histological reports, rejection episodes were retrospectively categorized as T cell-mediated rejection (TCMR, *n* = 4), antibody-mediated rejection (ABMR, *n* = 3) or mixed cellular/humoral rejection (*n* = 4). Detailed characteristics of rejection episodes are provided in Supplementary Table S9. Because of the limited number of rejection events in each subgroup, separate statistical analyses according to rejection phenotype were not feasible.

Biopsies were performed based on clinical indications rather than by protocol. As the timing of biopsy did not consistently coincide with immunophenotyping time points, recipients with rejection during the follow-up period were analyzed collectively.

### 3.6. A Group Comparisons at T0 and T12

To explore whether peripheral B-cell profiles differed between recipients who experienced rejection (Group 1) and those with stable graft function (Group 2), B-cell subpopulations were compared at pre-transplant baseline (T0) and at one year post-transplant (T12).

At T0, total B-cell frequencies and absolute numbers were comparable between Group 1 and Group 2 (frequency: 8.0% [IQR 6.1–11.2] vs. 8.1% [5.9–11.4], *p* = 0.8, absolute numbers: 105 [78–142] vs. 93 [71–128] cells/µL, p = 0.7). Group 1 showed numerically higher memory B-cell frequencies and numbers (frequency: 25% [21–33] vs. 22% [15–35], p = 0.6, absolute numbers: 23 [12–43] vs. 20 [13–32] cells/µL, *p* = 0.7), as well as with relatively lower naïve B-cell frequencies despite higher absolute numbers. tBregs and plasmablasts were also numerically higher in Group 1 at baseline. However, none of these differences reached statistical significance.

By T12, more pronounced differences were observed between groups, although most comparisons remained not statistically significant, Table S10. Compared with stable recipients, Group 1 demonstrated lower absolute numbers of total B cells (a 24.5% reduction) and naïve B cells (a 38% reduction), accompanied by reductions in relative frequencies (a 9% and a 16% reduction, respectively). In contrast, total memory B cells and mBregs were higher in group 1 in both frequency and absolute number. tBregs and plasmablasts were markedly reduced, approaching depletion in several recipients. Consistent with these shifts, the naïve-to-memory B-cell ratio was lower in group 1 at T12 (a 26.3% reduction in frequency and 40.9% reduction in absolute numbers).

Overall, pre-transplant B-cell profiles were unable to distinguish between recipients who later developed rejection and those with stable graft function. However, rejection episodes during follow-up were associated with a shift towards a memory-dominant peripheral B-cell profile within one year post-transplant.

### 3.7. Class-Switched Memory B Cells and Discrimination of Rejection

**Figure 2:**
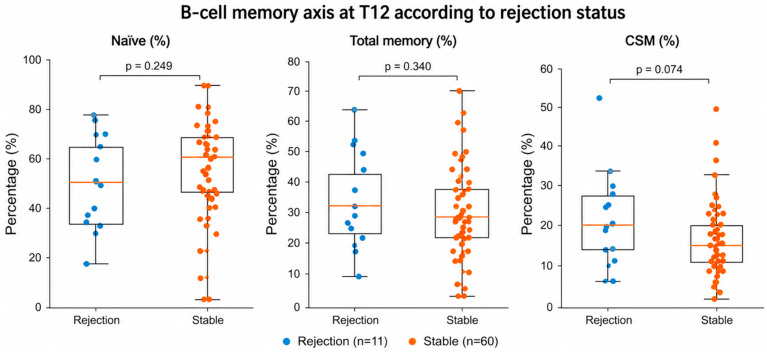
Distribution of key memory-related B-cell subsets at 12 months according to rejection status. Dot-and-box plots show naïve B cells, total memory B cells, and class-switched memory B cells in recipients with rejection episodes and stable graft function. Comparisons were performed using the Mann–Whitney U test.

Among the analyzed subpopulations, CSM B cells exhibited the most significant between-group difference at T12, with higher frequencies observed in recipients experiencing rejection compared to stable recipients. This difference almost reached statistical significance (median 21% [IQR 13–28] vs. 15% [IQR 10–20], *p* = 0.074, Figure 2).

**Figure 3:**
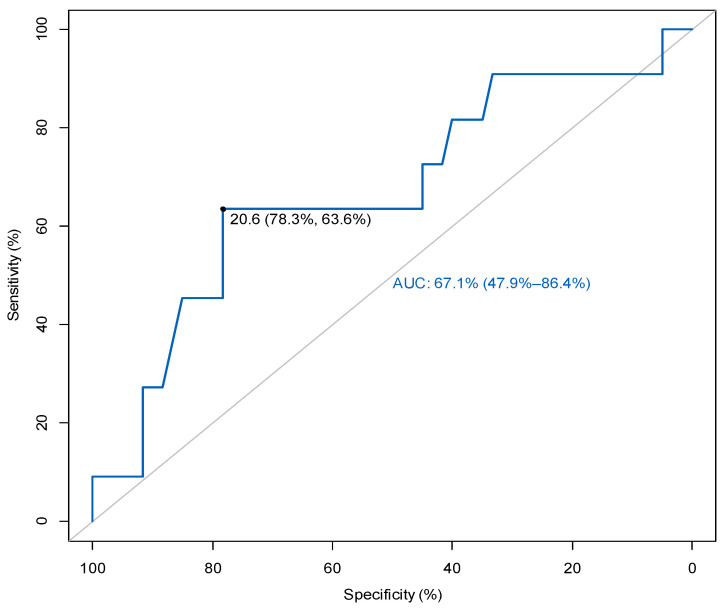
ROC curve for CSM B-cell frequency at T12 in relation to rejection episodes during the first year post-transplantation (AUC 0.671, 95% CI 0.479–0.864).

Given this pattern, we explored the discriminative performance of CSM frequency for distinguishing recipients with rejection episodes from those without. Receiver operating characteristic (ROC) analysis revealed only moderate discriminatory ability (area under the curve (AUC) 0.671, 95% confidence interval (CI) 0.479–0.864, Figure 3). A cut-off value of 20.6% yielded sensitivities of 63.6% and specificities of 78.3%. These findings suggest that elevated CSM frequencies may be associated with rejection episodes. Nevertheless, the observed discriminative performance was modest, which supports the idea that CSM frequency alone is unlikely to be a reliable independent clinical marker.

### 3.8. Memory-Dominant B-Cell Phenotypes Identified by Cluster Analysis

**Figure 4:**
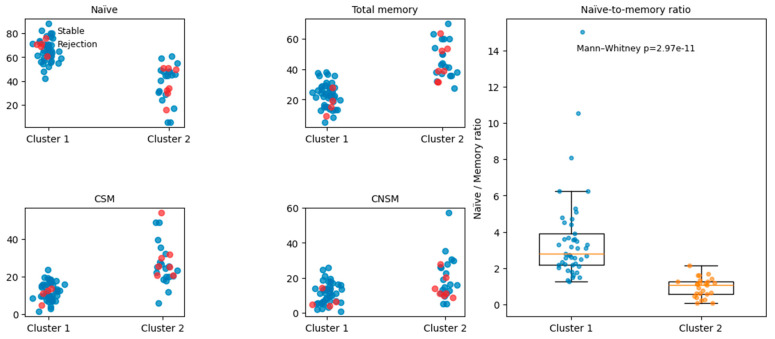
Cluster-associated distribution of peripheral B-cell subsets and naïve-to-memory ratio at 12 months post-transplantation. Dot plots show the frequencies of naïve B cells, total memory B cells, CSM B cells, and CNSM B cells according to hierarchical cluster membership. These variables contributed to cluster definition and are shown to illustrate the phenotypic differences between clusters. Each dot represents one patient, with red dots indicate recipients with rejection episodes and blue dots indicate stable recipients. The panel on the right illustrates the naïve-to-memory B-cell ratio across clusters. Comparisons of the ratio between clusters were performed using the Mann–Whitney U test.

To explore whether distinct peripheral immune phenotypes could be identified at one year, hierarchical clustering was performed using standardized frequencies of B-cell subpopulations at T12. Two clusters emerged: Cluster 1 (*n* = 48, 68%) and Cluster 2 (*n* = 23; 32%), Table S11. Rejection episodes were more frequent in Cluster 2 than Cluster 1 (30% vs. 8%; *p* < 0.05), Figure 4.

Immunophenotypically, Cluster 1 was characterized by higher frequencies of total B cells and naïve B cells, whereas Cluster 2 exhibited lower total and naïve B-cell frequencies with enrichment of memory subsets, including CSM and CNSM B cells (Table S11, Figure 4). Renal function at T12 did not differ significantly between cluster 1 and cluster 2 (median eGFR 59.4 [IQR 50.0–71.5] vs. 54.7 [41.5–68.8] mL/min/1.73 m^2^, Mann–Whitney U test, *p* = 0.197).

When compared with pre-transplant values, Cluster 1 demonstrated relatively modest post-transplant changes, whereas Cluster 2 showed reductions in total and naïve B cells alongside increases in memory subsets (Table S12). These findings indicate that a memory-dominant peripheral B-cell phenotype at one year was more frequently observed among recipients with rejection episodes, while a naïve-dominant phenotype was more commonly seen in recipients with stable graft function.

Because clustering was based on immune profiles measured at T12, these patterns may partly reflect the immunological consequences of earlier rejection episodes or their treatment rather than predictive biomarkers.

## 4. Discussion

A prospective analysis of circulating B cell subpopulations in kidney transplant recipients was conducted over a one-year period. Both the frequency and absolute numbers of these cells were evaluated in order to characterize post-transplant immune reconstitution and examine their relationship with clinical course, including rejection episodes [22,23,24,25]. In many cases these two measures were not aligned [26,27], as evidenced in the case of total B lymphocytes (CD19^+^) the frequency decreased over time while their absolute numbers showed a slight increase. Similarly, the frequency of naïve B cells decreased despite an increase in their absolute numbers, while memory B cells increased in both frequency and numbers [28,29,30,31]. These findings indicate the presence of time dependent changes in peripheral B-cell composition during the early post-transplant period and point to the importance of jointly considering proportional and quantitative measures when interpreting longitudinal immune dynamics.

The longitudinal evaluation further highlights the dynamic evolution of the peripheral B-cell compartment during the first post-transplant year, with recipients who experienced rejection tending to show lower naïve B-cell frequencies and relatively higher proportions of memory B-cell subsets over time compared with those with stable graft function. These finding support the presence of time dependent shifts in B cell composition during early immune reconstitution after transplantation.

tBregs showed decreased absolute numbers at all study time points, likely due to their immature characteristics and heightened sensitivity to immunosuppression [32,33,34]. Similar patterns were observed in both stable recipients and those who experienced rejection, consistent with previous reports in transplant recipients [33,34,35]. Importantly, these longitudinal patterns were similar after exclusion of recipients who received ATG induction therapy, indicating that the observed trends were not primarily driven by induction regimen. Although tBregs levels demonstrate positive correlations with eGFR at T6 and T12, these associations were modest and time dependent, suggesting only a limited relationship with graft function [32,36]. In contrast, mBregs remained stable over time [36], while plasmablast frequencies declined [9,27], a pattern that may reflect attenuation of early humoral immune activation under maintenance immunosuppression.

These relationships were modest and variable over time, and no other B-cell subsets showed consistent associations with graft function. Accordingly, B-cell phenotypes captures only part of the complex determinants influencing early post-transplant graft performance.

Several clinical variables including recipient age, dialysis vintage and donor type showed associations with B-cell subpopulations in univariate analysis, but these associations did not remain statistically significant after multivariate adjustment. The same applies to CIT. These findings suggest that the observed longitudinal B-cell dynamics are more likely to be related to post-transplant immune remodeling than to baseline clinical characteristics. Although age is known to influence B-cell composition, age-stratified baseline analyses revealed minimal differences in naïve and memory compartments in our cohort, suggesting that age did not materially account for the observed post-transplant immune patterns.

Comparative analyses at T0 and T12 demonstrated that pre-transplant peripheral B-cell profiles were broadly similar between recipients who later experienced rejection and those who maintained stable graft function. Although numerical differences were observed at baseline, including slightly higher memory B-cell representation and lower naïve B-cell frequencies in recipients who subsequently developed rejection, none reached statistical significance [37,38].

At T12, a more evident shift toward a memory-dominant profile was observed among recipients with rejection episodes, characterized by reduced naïve B-cell representation, increased memory B-cell proportions, and lower naïve-to-memory ratios and alterations in regulatory B cells and plasmablast. These findings are consistent with the overall longitudinal trends and likely reflect post-transplant immune adaptation associated with rejection events rather than pre-existing immunological risk.

The reduction in the naïve-to-memory ratio following rejection suggests an imbalance in B cell homeostasis and may reflect persistent immune activation or impaired regulation. Viewed longitudinally these findings indicate that assessment at T0 and T12 captures evolving immune patterns over time rather than stable baseline risk profiles.

Among individual subsets, class-switched memory B cells demonstrated the largest between-group difference at one year, with higher frequencies in recipients who experienced rejection. Although this association did not reach conventional statistical significance, ROC analysis suggested that a CSM frequency exceeding 20.6% was associated with rejection events during the first year post-transplant, with moderate sensitivity (78.3%). Expansion of CSM B cells has been linked to differentiation into antibody secreting cells, and may reflect ongoing humoral alloimmune activity [38,39]. However, given the modest sample size and retrospective grouping of rejection events, these finding should be interpreted as descriptive rather than predictive.

Important differences were observed when one year stable recipients were compared with long-term tolerant recipients characterized by stable graft function for over 25 years [40,41]. In our cohort, recipients with early graft stability had higher frequencies of naïve cells and lower frequencies of CSM B cells, contrary to the low naïve/high CSM profile reported in long-term tolerance.

These findings suggest that immunological stability in the early post-transplant period differs from the immune adaptations associated with durable long term tolerance. This comparison highlights that peripheral immune phenotypes observed during the first post-transplant year should be interpreted within a temporal framework, as they may represent transitional immune states rather than endpoints of immune adaptation.

These observations were supported by cluster analysis, which identified two immune phenotypes. The first—a naïve-dominant phenotype—was enriched among stable recipients, and characterized by higher frequencies of total B and naïve B cells, with lower frequencies of total memory, CSM and CNSM B cells. In contrast, the memory-dominant phenotype, encompassed most recipients who experienced rejection, exhibited reduced frequencies of total B cells and naïve B cells, alongside increased frequencies of total memory, CSM, and CNSM B cells. However, because the clustering analysis was based on B-cell frequencies measured one year after transplantation, these phenotypes should be interpreted as descriptive immune patterns associated with prior clinical events rather than as predictive signatures.

The present study is subject to several limitations, including the modest sample size inherent to a single-center cohort. This inevitably reduces statistical power and may explain why several differences between groups did not reach conventional significance thresholds. In addition, biopsies were performed for clinical indications and were not synchronized with immunophenotyping time points. The types of graft rejection—T-cell-mediated, antibody-mediated, or mixed—were not analyzed in detail, and the absence of peripheral T-cell subset phenotyping further limits the ability to interpret the B-cell findings within a broader immunological context. Functional assays assessing IL-10 production by tBregs or antibody-secreting B-cell activity were not performed. Moreover, gene expression analyses of B-cell-related molecules and pathways, such as BAFFR, BAFF, APRIL, or related receptors including TACI, were not evaluated, nor were soluble mediators produced by or associated with B cells assessed. Therefore, the present findings are based primarily on peripheral B-cell surface phenotyping and should be interpreted as descriptive immunophenotypic associations rather than direct functional evidence.

## 5. Conclusions

Peripheral B-cell profiles before and during the first year after transplantation appear to reflect ongoing immune remodeling, with memory-dominant patterns at one year being associated with rejection history. A memory-biased B cell profile marked by expansion of class-switched memory B cells was observed in recipients who experienced rejection, whereas naïve-dominant profiles were more frequently seen in those with early graft stability. In this context, the observed patterns indicate that longitudinal characterization of peripheral B cell immune landscapes, may offer insight into evolving post-transplant immune states. However, the modest discriminatory performance of individual subsets points to the limitations of peripheral B cell phenotyping as a standalone clinical tool. Future studies should validate these observations in larger multicenter cohorts incorporating synchronized functional, immunophenotypic, and histological assessments.

## Figures and Tables

**Table 1:** Patients demographics, clinical characteristics and characteristics related to transplantation [24].

Study Sample	*n* : 71
**Characteristics of recipients**	
Sex	Female: 20/Male: 51 28.17/71.83%
Age in years	48.5 (39–60)
Type of donors	Deceased brain death: 50 (70%) Living: 21 (30%)
Preemptive recipients	7 (9.86%)
**Dialysis patients candidates for transplantation**	
Type of dialysis	HD: 64 (81%) CAPD: 7 (19%)
Duration of dialysis (months)	87 (34–127)
**Distribution of primary cause of renal failure, *n***	
Polycystic kidney diseases	14 (19.7%)
Primary glomerulonephritis:	12 (17%)
IgA nephropathy	5 (7%)
Membranous nephropathy	3 (4.25%)
Focal segmental glomerulosclerosis	2 (2.83%)
Membranoproliferative glomerulonephritis	2 (2.83%)
Reflux nephropathy	6 (8.4%)
Diabetis melitus	6 (8.4%)
Nephrosclerosis/hypertension	8 (11.25%)
Urinary tract infections/stones	5 (7%)
Other	12 (17%)
Unknown	8 (11.25%)
**Information on Transplantation,** **Graft function**	
Delayed graft function	Yes: 21 (29.6%) No: 50 (70.4%)
Cold Ischemia Time (hours)	19.2 (4.6)
eGFR (mL/min/1.73 m^2^)	52 (36–89)
Recipients with Rejection	11 (15.5%)
**Induction therapy with**	
Basiliximab, *n* (%)	63 (88.7%)
Anti-thymocyte globulin, *n* (%)	8 (11.3%)

**Table 2:** Changes in the frequency of B lymphocytes and its subpopulations at T0, T3, T6 and T12.

Frequency of Cell Populations (%)	T0 ^1^	T3 ^1^	T6 ^1^	T12 ^1^	*p* ^2^	Post Hoc ^3^ Comparison
B lymphocytes	8 (6, 11.3)	8.9 (5.7, 13)	7.4 (5.3, 10)	6.7 (5.2, 9.8)	<0.001	t3–t6 *p* = 0.002 t3–t12 *p* = 0.026
Naive	61.4 (51.9, 74.5)	60 (47.5, 75.2)	60 (42.7, 69.3)	59.3 (48, 68.1)	ns	t0–t12 *p* = 0.29 t3–t12 *p* = 0.088
Total memory	24.6 (15.1, 34.1)	25.7 (15.2, 37.3)	27 (13.5, 38)	28.1 (21.4, 38)	0.05	t0–t12 *p* = 0.009
Class switched memory	13.4 (8.9, 20.4)	12.8 (7.9, 19.7)	13.7 (8.4, 18.7)	15.9 (9.9, 21.5)	0.01	t3–t12 *p* = 0.019
Class non switched memory	8.5 (4, 13.7)	10.2 (4.8, 15.9)	9.4 (4, 16.7)	12.8 (6.7, 17.5)	0.02	t0–t12 *p* = 0.008
Plasmablasts	0.1 (0, 1.2)	0 (0, 1)	0 (0, 0.6)	0 (0, 0.5)	0.02	t0–t6 *p* = 0.031
tBregs	1.5 (0.3, 3.5)	0.8 (0.1, 3)	0.2 (0, 1)	0 (0, 1)	<0.001	t0–t12 *p* < 0.001 t0–t6 *p* < 0.001 t3–t6 *p* = 0.02 t3–t12 *p* = 0.015
mBregs	2 (0.2, 4.6)	3.1 (0.4, 6.5)	1.8 (0.1, 3.6)	2 (0.3, 5.9)	ns	t3–t6 *p* = 0.042

Median ^1^ (IQR); Friedman ^2^ test; Wilcoxon ^3^ signed-rank test. ns: not significant.

**Table 3:** Correlation between B cell subpopulations and eGFR at T6 and T12. Spearman correlation coefficients (r) and corresponding *p* values for associations between circulating B cell subpopulations and eGFR at T6 and T12 post transplantation. Statistically significant associations (<0.05) are shown in bold.

Cell Populations		eGFR/T6	eGFR/T12
Β lymphocytes % (CD19+)	*r*	−0.409	0.350
	*p*-value	0.16	0.39
Β lymphocytes/μL (CD19+)	*r*	0.23	0.64
	*p*-value	0.63	0.337
Naïve B lymphocytes % (CD19+IgD+CD27-)	*r*	0.37	0.50
	*p*-value	0.39	0.16
Naïve B lymphocytes/μL (CD19+IgD+CD27-)	*r*	0.352	−0.29
	*p*-value	0.66	0.37
Total memory B lymphocytes %	*r*	−0.37	0.32
	*p*-value	0.71	0.91
Total memory B lymphocytes/μL	*r*	−0.02	0.07
	*p*-value	0.570	0.860
Class switch memory %, (CD19+IgD-CD27+),	*r*	−0.18	0.13
	*p*-value	0.49	0.91
Class switch memory/μL, (CD19+IgD-CD27+)	*r*	0.04	−0.27
	*p*-value	0.82	0.650
Class non switch memory %, (CD19+IgD+CD27+)	*r*	0.060	−0.079
	*p*-value	0.52	0.690
Class non switch memory/μL, (CD19+IgD+CD27+)	*r*	0.04	0.022
	*p*-value	0.31	0.34
Plasmablasts %	*r*	**−0.17**	−0.15
	*p*-value	**0.012**	0.1
Plasmablasts/μL	*r*	**−0.29**	0.12
	*p*-value	**0.009**	0.2
tBregs % CD19+CD24++CD38++	*r*	**0.27**	**0.653**
	*p*-value	**<0.001**	**0.04**
tBregs/μL CD19+CD24++CD38++	*r*	**0.31**	**0.11**
	*p*-value	**<0.001**	>0.05
mBregs % CD19+CD24++CD27+	*r*	0.05	0.07
	*p*-value	0.23	0.66
mBregs/μL CD19+CD24++CD27+	*r*	0.19	0.21
	*p*-value	0.39	0.18

## Data Availability

Upon request, the corresponding author can provide the datasets used and analyzed in this study.

## Supplementary Materials

The following supporting information can be downloaded at: https://www.mdpi.com/article/10.3390/jcm15114064/s1, Figure S1: Gating strategy for B cell subpopulations. Representative flow cytometric data. CD45 and side scatter used for lymphocyte gating. Total B cells are CD19+ cells, which are then analyzed for tBregs and mBregs cells. Frequencies of each subset were expressed as percentages of total circulating B cells (CD19^+^); Table S1: Antibodies used to identify B cell subpopulations; Figure S2: Longitudinal trajectories of circulating B-cell subsets according to rejection status. Distribution of naïve B cells, total memory B cells, switched memory B cells, and transitional regulatory B cells (tBregs) at pre-transplant (T0) and at 3, 6, and 12 months after transplantation (T3, T6, T12). Recipients were stratified according to the occurrence of rejection during follow-up. Boxplots represent median and interquartile range with individual patient values overlaid. *p*-values were calculated using the Wilcoxon rank-sum (Mann–Whitney U) test; Table S2: Changes in the absolute numbers of B lymphocytes and its subpopulations at T0, T3, T6 and T12; Table S3: Sensitivity analysis of longitudinal B-cell subset kinetics excluding recipients treated with ATG (*n* = 63); Table S4: Median frequencies and interquartile ranges (IQR) of selected B-cell subsets at T0, T3, T6 and T12 in recipients with stable graft function and those who experienced rejection. Group comparisons at each timepoint were performed using the Wilcoxon rank-sum (Mann–Whitney U) test; Table S5: Results of the univariate analysis of the frequencies of B lymphocytes, naive B cells, total memory, class switched memory, class non switched memory in relation to the age of recipients, the type of donor, cold ischemia time, delayed graft function and dialysis vintage. β: beta coefficient; *p*-value, values of *p* ≤ 0.05 were considered statistically significant; *n*: number of participants in the study. The number of participants differs in the dialysis vintage parameter because the preemptive candidates were not included; Table S6: Results of the univariate analysis of the absolute numbers of B lymphocytes, naive B cells, total memory, class switched memory, class non switched memory in relation to the age of recipients, the type of donor, cold ischemia time, delayed graft function and dialysis vintage. β: beta coefficient; *p*-value, values of *p* ≤ 0.05 were considered statistically significant, n: number of participants in the study. The number of participants differs in the dialysis vintage parameter because the preemptive candidates were not included; Table S7: Baseline (T0) B-cell subsets according to age; Table S8: Characteristics of patients in Groups 1 and 2. Group 1: recipients with rejection, Group 2: recipients without rejection; Table S9: Clinical, histological, and treatment characteristics of rejection episodes during the first post-transplant year; Table S10. Frequency and absolute counts of circulating B-cell subpopulations at T12 in recipients with rejection episodes (Group 1) and stable recipients (Group 2); Table S11: The differences in the median (interquartile range [IQR]) of the frequencies of the B cell subpopulations in the two clusters; Table S12: Comparison of variables before (T0) and one year after transplantation (T12) in patients within each cluster.
