# Supplementary material for: Early Post-Transplant Peripheral B-Cell Profiles in Kidney Transplant Recipients: Clinical Associations and Limitations
Source: J Clin Med. 2026 May 24;15(11):4064. doi: 10.3390/jcm15114064 (PMC13258135; doi:10.3390/jcm15114064)
Supplement: Supplementary file 1 [file jcm-15-04064-s001.zip › jcm-4257627-supplementary.pdf]

Supplementary Methods

Whole blood was stained with fluorochrome-conjugated monoclonal antibodies (Table S1). Samples were analyzed on an 8-colour Navios flow cytometer (Beckman Coulter, Marseille, France). Data were acquired with Kaluza software and processed according to established gating strategies [21,22].
For regression analyses (Tables S3–S4), significance was set at $p \leq 0.05$.
For cluster comparisons (Tables S7–S9), a multiple-testing threshold of $p < 0.005$ was used. Clusters 1–2 were derived by unsupervised clustering, rejection status was compared separately

Flow Cytometric Analysis of B Cell Subsets

Gating procedure

-Doublets were excluded by plotting forward scatter height versus area.

Monocytes, lymphocytes, and granulocytes were identified by forward/side scatter characteristics. B cell subset gating was performed on lymphocytes, Figure S1 with the following definitions:

Naïve B cells: $CD19^{+}IgD^{+}CD27^{-}$, Memory B cells: $CD19^{+}CD27^{+}$,Class-switched memory (CSM): $CD19^{+}IgD^{-}CD27^{+}$,Class-non-switched memory (CNSM): $CD19^{+}IgD^{+}CD27^{+}$

Regulatory B cells (Bregs):Transitional Bregs (tBregs): $CD19^{+}CD24^{++}CD38^{++}$, Memory Bregs (mBregs): $CD19^{+}CD24^{++}CD27^{+}$, Figure S1.

Plasmablasts: $CD27^{++}CD38^{++}IgD^{+}/^{-}$.

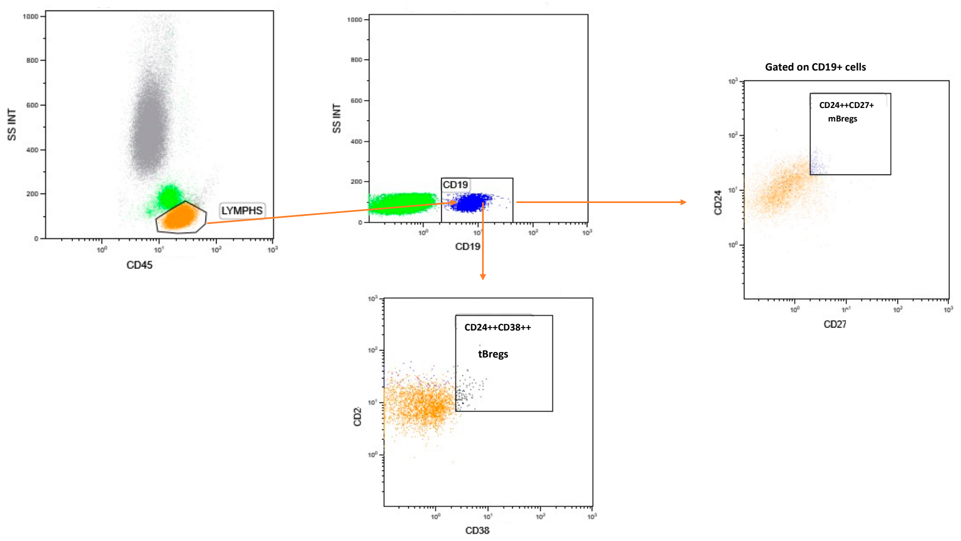

Figure S1: Gating strategy for B cell subpopulations. Representative flow cytometric data. CD45 and side scatter used for lymphocyte gating. Total B cells are CD19+ cells,

which are then analyzed for tBregs and mBregs cells. Frequencies of each subset were expressed as percentages of total circulating B cells ($CD19^+$).

Table S1: Antibodies used to identify B cell subpopulations

**Conjugated Antibodies (clones)**	**Manufacturer**
anti-CD19 PC5.5 clone J3-119.	Beckman, Coulter
anti -CD27 PE-Dylight 594. clone LT27	EXBIO, Praha SA
anti-IgD PerCP-efluor 710. clone IA6-2	ThermoScientific LSG
anti-CD38-PB clone LS198-4-3	Beckman, Coulter
anti-CD24- APC-Cy7 clone SN3	EXBIO, Praha SA
anti-CD45-PC7 clone J33	Beckman, Coulter

Table S2: Changes in the absolute numbers of B lymphocytes and its subpopulations at T0, T3, T6 and T12

**Absolute numbers of cell populations (cells/μL)**	**T0** [1]	**T3** [1]	**T6** [1]	**T12** [1]	***p*** [2]	**Post Hoc** [3] **Comparison**
**B lymphocytes**	93 (58.8, 144.7)	100 (48.4, 170)	86 (53.6, 136.7)	101 (61.2, 163.5)	0.04	Ns
**Naïve**	52 (29.7, 101)	53 (25.2, 121.9)	42 (27.7, 76.2)	62 (27.5, 100.4)	ns	
**Total memory**	21 (12.4, 33.6)	22 (10.7, 40)	18 (8.9, 34.1)	30 (17.9, 41.8)	0.03	t0-t12 p = 0.032<br>t6-t12 p = 0.028
**Class switched memory**	12 (7.6, 19.6)	12 (6, 19.2)	11 (5.1, 17.4)	17 (9, 22.6)	0.01	t3-t12 p = 0.019
**Class non switched memory**	8 (3.2, 13.8)	8 (3.3, 19.7)	5 (3, 14.5)	13 (6.5, 20.7)	0.001	t0-t12 p = 0.002<br>t6-t12 p = 0.013
**Plasmablasts**	0.05 (0, 0.3)	0 (0, 0.1)	0 (0, 0.1)	0 (0, 0.2)	0.03	t0-t6 p = 0.045

**tBregs**	1.2 (0.2, 4.2)	0.5 (0.1, 3.7)	0.1 (0, 0.9)	0 (0, 1.2)	<0.001	t0-t6 p < 0.001<br>t0-t12 p < 0.001<br>t3-t6 p = 0.004<br>t3-t12 p = 0.002
**mBregs**	2 (0.1, 5.1)	3 (0.4, 8.3)	1 (0.1, 3)	2 (0.2, 4.5)	Ns	ns

Median [1] (IQR); Friedman [2] test; Wilcoxon [3] signed- rank test.

Table S3: Sensitivity analysis of longitudinal B-cell subset kinetics excluding recipients treated with ATG (n = 63).

B-cell subset	T0 Median (IQR)	T3 Median (IQR)	T6 Median (IQR)	T12 Median (IQR)	p value (Friedman)
Total B cells (%)	8.0 (6.0–11.0)	8.8 (5.6–13.0)	7.3 (5.2–10.0)	6.5 (5.1–9.7)	**0.002**
Naïve B cells (%)	61.2 (51.7–74.0)	60.0 (47.0–75.0)	59.6 (42.5–69.0)	58.9 (47.5–68.0)	**0.041**
Total memory (%)	24.5 (15.0–34.0)	25.5 (15.0–37.0)	26.8 (13.0–37.5)	28.0 (21.0–38.0)	**0.031**
Switched memory (%)	13.3 (8.8–20.0)	12.6 (7.8–19.5)	13.5 (8.2–18.5)	15.7 (9.8–21.3)	**0.023**
Non-switched memory (%)	8.3 (4.0–13.5)	10.0 (4.7–15.7)	9.2 (4.0–16.5)	12.5 (6.5–17.3)	0.051
Plasmablasts (%)	0.10 (0–1.2)	0 (0–1.0)	0 (0–0.6)	0 (0–0.5)	**0.017**
tBregs (%)	1.5 (0.3–3.4)	0.8 (0.1–3.0)	0.2 (0–1.0)	0 (0–1.0)	**<0.001**
mBregs (%)	2.0 (0.2–4.5)	3.0 (0.4–6.3)	1.7 (0.1–3.5)	2.0 (0.3–5.7)	0.073

Values are presented as median (interquartile range)**.**
p-values calculated using Friedman test for repeated measures.

Table S4: Median frequencies and interquartile ranges (IQR) of selected B-cell subsets at T0, T3, T6 and T12 in recipients with stable graft function and those who experienced rejection. Group comparisons at each timepoint were performed using the Wilcoxon rank-sum (Mann–Whitney U) test.

B-cell subset	Time	Stable Median (IQR)	Rejection Median (IQR)	p-value
**Naïve B cells**	T0	61.45 (54.00–68.20)	57.60 (48.30–63.50)	0.956
	T3	61.65 (55.10–67.90)	57.10 (46.80–63.40)	0.781
	T6	60.10 (52.80–66.40)	41.30 (32.70–55.90)	0.409
	T12	60.80 (54.60–67.10)	50.70 (41.20–58.90)	0.249
**Total memory B cells**	T0	21.90 (17.80–27.40)	24.90 (18.20–30.70)	0.645
	T3	25.35 (20.10–31.40)	26.90 (21.30–34.10)	0.937

	T6	26.90 (21.60–32.80)	33.50 (25.40–38.20)	0.499
	T12	27.75 (22.10–33.40)	31.70 (25.90–36.80)	0.340
**Switched memory B cells**	T0	13.30 (9.40–18.10)	17.20 (11.80–21.60)	0.781
	T3	12.10 (8.90–16.20)	17.60 (12.40–23.50)	0.201
	T6	13.35 (9.10–17.70)	17.00 (11.90–22.40)	0.378
	T12	15.35 (10.70–19.80)	20.60 (14.30–25.10)	0.074
**Transitional Bregs**	T0	1.50 (0.70–2.30)	2.40 (1.10–3.50)	0.356
	T3	0.70 (0.30–1.40)	1.80 (0.90–2.70)	0.257
	T6	0.20 (0.00–0.80)	0.00 (0.00–0.40)	0.810
	T12	0.10 (0.00–0.60)	0.00 (0.00–0.30)	0.375

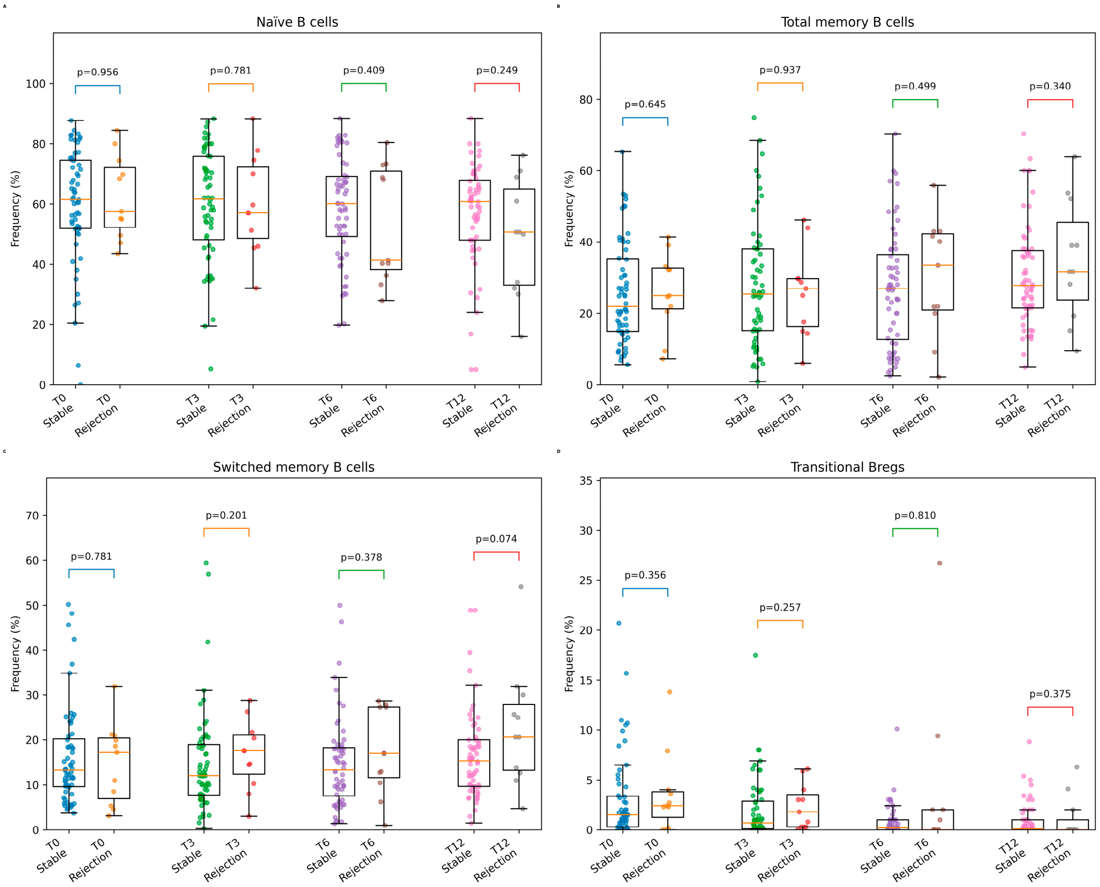

Figure S2: Longitudinal trajectories of circulating B-cell subsets according to rejection status.

Distribution of naïve B cells, total memory B cells, switched memory B cells, and transitional regulatory B cells (tBregs) at pre-transplant (T0) and at 3, 6, and 12 months after transplantation (T3, T6, T12). Recipients were stratified according to the occurrence of rejection during follow-up. Boxplots represent median and interquartile range with individual patient values overlaid. P-values were calculated using the Wilcoxon rank-sum (Mann–Whitney U) test.

Table S5: Results of the univariate analysis of the frequencies of B lymphocytes, naive B cells, total memory, class switched memory, class non switched memory in relation to the age of recipients, the type of donor, cold ischemia time, delayed graft function and dialysis vintage. β: beta coefficient; *p*-value, values of $p \leq 0.05$ were considered statistically significant; n: number of participants in the study. The number of participants differs in the dialysis vintage parameter because the preemptive candidates were not included.

	**Univariate Regression**					**Multivariate Regression**			
**frequency of cell population**				**95% confidence interval**				**95% confidence interval**	
**B lymphocytes**	**β**	**n**	**p**	**lower**	**upper**	**β**	**p**	**lower**	**upper**
Age of the recipient	-0.12	71	0.007	-0.2	-0.03	-0.05	0.7	-0.28	0.19
Type of donor (deceased/living)	-0.42	71	0.7	-6.2	-1.4				
Cold ischemia time, CIT	-0.31	71	0.8	-0.1	0.12				
Delayed graft function, DGF	-1.8	71	0.11	-4.1	0.41	-0.69	0.8	-5.4	4
Dialysis vintage	-0.02	64	0.1	-0.04	0.00	-0.02	0.4	-0.08	0.03
**Naïve B cells**									
Age of the recipient	-0.24	71	0.2	-0.59	0.11				
Type of donor (deceased/living)	-0.24	71	0.5	-12	5.9				
Cold ischemia time, CIT	-0.09	71	0.2	-0.59	0.11				
Delayed graft function, DGF	-0.16	71	0.08	-2	0.13				
Dialysis vintage	-0.02	64	0.6	-0.1	0.06				
**Total memory**									
Age of the recipient	0.25	71	0.093	-0.04	0.54				
Type of donor (deceased/living)	3.8	71	0.3	-3.8	11				
Cold ischemia time, CIT	-0.14	71	0.4	-0.51	0.23				
Delayed graft function, DGF	-0.08	71	>0.9	-7.7	7.5				
Dialysis vintage	0.03	64	0.4	-0.04	0.10				
**Class-switched memory B cells**									
Age of the recipient	0.19	71	0.074	-0.02	0.40				
Type of donor (deceased/living)	-0.69	71	0.8	-6.2	4.8				
Cold ischemia time, CIT	0.05	71	0.7	-0.21	0.32				

Delayed graft function, DGF	2.6	71	0.3	-2.8	8				
Dialysis vintage	0.01	64	0.6	-0.04	0.06				
**Class-non switched memory B cells**									
Age of the recipient	0.03	71	0.7	-0.15	0.22				
Type of donor (deceased/living)	-0.69	71	0.8	-6.2	4.8	3.7	0.4	-6.1	14
Cold ischemia time, CIT	-0.19	71	0.1	-0.42	0.03	-0.01	>0.9	-0.48	0.47
Delayed graft function, DGF	-3.1	71	0.2	-7.8	1.6	-1.9	-6.9	3	0.4
Dialysis vintage	0.01	64	0.4	-0.02	0.05				
**plasmablasts**									
Age of the recipient	-0.03	71	0.057	-0.06	0.00	-0.01	0.5	-0.06	0.03
Type of donor (deceased/living)	0.55	71	0.2	-0.22	1.3	0.47	0.6	-1.4	2.3
Cold ischemia time, CIT	-0.02	71	0.3	-0.06	0.02	0.01	0.8	-0.07	0.09
Delayed graft function, DGF	-0.04	71	>0.9	-0.82	0.74				
Dialysis vintage	-0.01	64	0.048	-0.01	0.00	0.00	0.4	-0.01	0.01
**tBregs**									
Age of the recipient	0.28	71	0.5	-0.62	1.2				
Type of donor (deceased/living)	0.00	71	0.7	-0.01	0.01				
Cold ischemia time, CIT	-0.36	71	0.4	-1.3	0.55				
Delayed graft function, DGF	0.01	71	0.5	-0.03	0.06				
Dialysis vintage	0.00	64	0.8	-0.03	0.04				
**mBregs**									
Age of the recipient	0.02	71	0.6	-0.06	0.09				
Type of donor (deceased/living)	-1.4	71	0.2	-3.3	0.61	-0.77	0.6	-3.7	2.2
Cold ischemia time, CIT	0.05	71	0.3	-0.05	0.14				
Delayed graft function, DGF	0.97	71	0.3	-0.99	2.9				
Dialysis vintage	0.00	64	0.8	-0.03	0.04				

Table S6: Results of the univariate analysis of the absolute numbers of B lymphocytes, naive B cells, total memory, class switched memory, class non switched memory in relation to the age of recipients, the type of donor, cold ischemia time, delayed graft function and dialysis vintage. β: beta coefficient; *p*-value, values of $p \leq 0.05$ were

considered statistically significant, n: number of participants in the study. The number of participants differs in the dialysis vintage parameter because the preemptive candidates were not included.

	**Univariate Regression**					**Multivariate Regression**			
**Absolute numbers (cells/μL)**				**95% confidence interval**				**95% confidence interval**	
**B lymphocytes**	**β**	**n**	**p**	**lower**	**upper**	**β**	**p**	**lower**	**upper**
Age of the recipient	-2.7	71	0.018	-5.0	-0.48	-1.2	0.4	-4.3	1.9
Type of donor (deceased/living)	50	71	0.1	-9.9	111	-11	0.9	-6.8	5.4
Cold ischemia time, CIT	-2.5	71	0.091	-5.4	0.41	-0.71	0.8	-6.8	5.4
Delayed graft function, DGF	-35	71	0.2	-95	25	-28	0.4	-93	36
Dialysis vintage	-0.67	64	0.015	-1.2	-0.13	-0.49	0.2	-1.3	0.31
**Naïve B cells**									
Age of the recipient	-1.7	71	0.044	-3.4	-0.05	-0.84	0.5	-3.1	2.6
Type of donor (deceased/living)	25	71	0.3	-20	70				
Cold ischemia time, CIT	-1.3	71	0.2	-3.5	0.82	0.24	0.9	-3.1	2.6
Delayed graft function, DGF	-21	71	0.4	-65	24				
Dialysis vintage	-0.47	64	0.023	-0.87	-0.07	-0.33	0.2	-0.89	0.23
**Total memory**									
Age of the recipient	-0.57	71	0.017	-1.0	-0.10	-0.09	0.8	-0.65	0.47
Type of donor (deceased/living)	19	71	0.002	7.4	31	6.8	0.6	-18	32
Cold ischemia time, CIT	-0.85	71	0.004	-1.4	-0.27	-0.06	>0.9	-1.2	1
Delayed graft function, DGF	-13	71	0.031	-25	-1.2	-7.8	0.2	-19	3.8
Dialysis vintage	-0.08	64	0.12	-0.18	0.02	-0.03	0.7	-0.17	0.11
**Class-switched memory B cells**									
Age of the recipient	-0.34	71	0.030	-0.65	0.03	-0.13	0.6	-0.55	0.3
Type of donor (deceased/living)	8.3	71	0.045	0.2	16	5.3	0.6	-13	24
Cold ischemia time, CIT	-0.37	71	0.058	-0.76	0.01	-0.03	>0.9	-0.86	0.8
Delayed graft function, DGF	-6.4	71	0.12	-14	1.7	-4.8	0.3	-14	4
Dialysis vintage	-0.07	64	0.064	-0.14	0.00	-0.02	0.7	-0.13	0.08
**Class-non switched memory B cells**									
Age of the recipient	-0.31	71	0.018	-0.57	-0.05	-0.05	0.7	-0.29	0.2
Type of donor (deceased/living)	11	71	0.002	4	17	-1.5	0.8	-12	9.3
Cold ischemia time, CIT	-0.47	71	0.005	-0.78	-0.15	-0.03	>0.9	-0.51	0.45

Delayed graft function, DGF	-8.1	71	0.018	-15	-1.4	-4.6	0.076	-9.6	0.49
Dialysis vintage	0.03	64	0.2	-0.07	0.02	-0.02	0.4	-0.09	0.04
**plasmablasts**									
Age of the recipient	-0.01	71	0.052	-0.02	0.19				
Type of donor (deceased/living)	0.11	71	0.3	-0.10	0.32				
Cold ischemia time, CIT	0.0	71	0.5	-0.01	0.01				
Delayed graft function, DGF	-0.01	71	>0.9	-0.22	0.19				
Dialysis vintage	0.01	64	0.4	0.01	0.00				
**tBregs**									
Age of the recipient	0.00	71	0.7	-0.03	0.02				
Type of donor (deceased/living)	-0.08	71	0.8	-0.83	0.66				
Cold ischemia time, CIT	0.01	71	0.5	-0.02	0.05				
Delayed graft function, DGF	0.01	71	0.8	-0.64	0.83				
Dialysis vintage	0.00	64	>0.9	-0.01	0.01				
**mBregs**									
Age of the recipient	-0.09	71	0.04	-0.18	0.0				
Type of donor (deceased/living)	-0.82	71	0.5	-0.1	0.12				
Cold ischemia time, CIT	0.01	71	0.9	-0.1	0.12				
Delayed graft function, DGF	-0.08	71	>0.9	-2.3	2.2				
Dialysis vintage	-0.01	64	0.3	-0.03	0.01				

Age-stratified baseline analysis

Because age may influence the balance between naïve and memory B-cell compartments, baseline B-cell subpopulations were additionally analyzed according to age strata defined by the cohort median age (48 years). No significant differences were observed between younger and older recipients for total B cells, naïve B cells, or memory B-cell subsets. However, older recipients exhibited higher frequencies of mBregs at baseline ($p = 0.015$). Overall, age did not appear to substantially influence the distribution of naïve or memory B-cell compartments at baseline.

Age stratification:

Median age: 48 years

Younger group: ≤48 years (n = 35)

Older group: >48 years (n = 34)

Table S7: Baseline (T0) B-cell subsets according to age

Subset	Younger median	Older median	p value
Total B cells	8.9	7.0	0.12
Naïve B cells	61.5	60.5	0.96

Total memory	22.7	24.8	0.97
Switched memory	13.5	13.3	0.60
Non-switched memory	9.1	7.9	0.44
Plasmablasts	0.20	0.15	0.92
tBregs	1.1	1.75	0.27
**mBregs**	**0.9**	**2.85**	**0.015**

No significant age effect was observed for most B-cell subsets at baseline. mBregs were significantly higher in older recipients (p = 0.015). Naïve and memory compartments were not significantly influenced by age in this cohort

Table S8: Characteristics of patients in Groups 1 and 2. Group 1: recipients with rejection, Group 2 : recipients without rejection.

**Characteristc**	**Total** **n:71**	**Group 1** **n:11**	**Group2** **n:60**	***p*** **Group 1 vs Group 2**
**Age**	49 (40, 57)	49 (38.25, 57)	48 (43, 52)	0.968
**sex (female/male)**	19/52	3/8	16/44	1.000
**Type of donation deceased/living**	50/21	7/4	43/17	0.721
**Patients with DGF**	21/71	6/11	15/60	0.072
**CIT**	35/71	7/11	28/60	0.6

Table S9: Clinical, histological, and treatment characteristics of rejection episodes during the first post-transplant year

**Patient**	**Month**	**Biopsy indication**	**Histology report**	**Treatment**	**ATG therapy**
1	4	Creatinine rise	Mixed cellular/humoral	Steroids, ATG, plasmapheresis	No
2	6 after sampling	Creatinine rise/dysfunction	Mixed cellular/humoral	Steroids, ATG	Yes
3	6 after sampling	Creatinine rise	T-cell mediated	ATG	Yes
4	3	Creatinine rise	T-cell mediated	ATG	No
5	7	Creatinine rise/ dysfunction	T-cell mediated	ATG	Yes
6	6	Creatinine rise/ dysfunction	Mixed cellular/humoral	Steroids, ATG, plasmapheresis	Yes
7	7	dysfunction	Mixed cellular/humoral	Steroids, ATG	No
8	3	Creatinine rise	ABMR	plasmapheresis, corticosteroids and	No

				intravenous immunoglobulin.	
9	7	Creatinine rise	ABMR	plasmapheresis, corticosteroids and intravenous immunoglobulin.	No
10	7	dysfunction	T-cell mediated	ATG	Yes
11	8	Creatinine rise/ dysfunction	ABMR	plasmapheresis, corticosteroids and intravenous immunoglobulin.	No

Table S10. Frequency and absolute counts of circulating B-cell subpopulations at T12 in recipients with rejection episodes (Group 1) and stable recipients (Group 2).

**Cell population at T12 frequency % # absolute numbers (cells/µL)**	**Group 1 n = 11 Median (IQR)**	**Group 2, n = 60 Median (IQR)**	**p-value Wilcoxon rank sum test**
% B-lymphocytes	6.2 (5.6, 9.1)	6.8 (5.1, 10.0)	0.9
# B-lymphocytes	77 (61, 142)	102 (60, 182)	0.7
% naïve	51 (33, 65)	61 (48, 68)	0.2
# naïve	41 (22, 72)	66 (30, 103)	0.3
% total memory	32 (24, 46)	28 (21, 38)	0.3
# total memory	31 (20, 45)	30 (16, 42)	0.6
% switched memory	21 (13, 28)	15 (10, 20)	0.074
# switched memory	15 (13, 29)	17 (9, 22)	0.3
% non-switched memory	11 (8, 14)	13 (7, 18)	0.5
# non-switched memory	15 (8, 16)	13 (6, 21)	0.7
% plasmablasts	0.01 (0.00, 0.60)	0.00 (0.00, 0.50)	0.9
# plasmablasts	0.03 (0.00, 0.25)	0.00 (0.00, 0.13)	0.6
% tBregs	0.1 (0.00, 1.00)	0.10 (0.00, 1.00)	0.4
# tBregs	0.1 (0.00, 0.39)	0.07 (0.00, 1.30)	0.3
% mBregs	3.9 (0.7, 5.9)	2.0 (0.3, 5.2)	0.8
# mBregs	3.0 (0.4, 5.2)	1.6 (0.2, 4.3)	0.7

Table S11: The differences in the median (interquartile range [IQR]) of the frequencies of the B cell subpopulations in the two clusters.

Model Variables of the Model (% cells)	Cluster 1 n = 48 (68%) Median (IQR)	Cluster 2 n = 23 (32%) Median (IQR)	*p*- value significant < 0.005	Statistical method
**B lymphocytes**	8.3 (6.1, 11.2)	5.6 (4.3, 6.6)	p < 0.005	Linear regression
**Naïve B cells,**	64.3 (59.2, 70.8)	44.5 (29.5, 48)	P < 0.005	Linear regression
**Total memory B cells**	24 (16.3, 28.7)	41.1 (37.6, 57)	p < 0.005	Linear regression
**Class-switched memory B cells,**	12.7 (8.7, 16.7)	26.9 (20.6, 31)	p < 0.005	Linear regression
**Class non-switched memory B cells**	11.6 (6.2, 15.3)	14 (11.1, 26.2)	p < 0.005	Linear regression
**Plasmablasts**	0 (0, 0.5)	0 (0, 0.8)	Ns	Linear regression
**tBregs**	0.1 (0, 1.0)	0 (0, 0.1)	Ns	Linear regression
**mBregs**	2 (0.2, 5.0)	2 (0.6, 5.9)	Ns	Linear regression

Comparisons of variables at T0 and T12 in patients within each cluster were performed for those that showed statistical significance in Table S11.

Table S12: Comparison of variables before (T0) and one year after transplantation (T12) in patients within each cluster.

**Cluster 1 T0-T12**			
Variable	**Median (IQR)**	**W-Value**	***p*-value**
**B lymphocytes T0**<br>**B lymphocytes T12**	9.8 (6.2, 13)<br>8.3 (6.1, 9.2)	600	0.906
**Naïve B cells T0**<br>**Naïve B cells T12**	69 (56.6, 77.1)<br>64.3 (59.2,70.8)	485	0.293
**Total memory B cells T0**<br>**Total memory B cells T12**	17.7 (12.7, 25.7)<br>24 (16.3, 28.7)	660.5	0.460
**Class switched memory B cells T0**<br>**Class switched memory B cells T12**	11.2 (6.2, 14.1)<br>12.7 (8.7, 16.7)	729	0.149
**Class non switched memory T0**<br>**Class non switched memory T12**	6.4 (3, 11.4)<br>11.6 (6.2, 15.3)	484.5	0.290

Significance threshold: p < 0.005 (multiple testing correction)

**Cluster 2 T0-T12**			
Variable	**Median (IQR)**	**W-Value**	***p*-value**
**B lymphocytes T0**<br>**B lymphocytes T12**	6.8 (6, 8.3)<br>5.6 (4.3, 6.6)	226.5	0.007

**Naïve B cells T0**<br>**Naïve B cells T12**	50.4 (42.2, 60.8)<br>44.5 (29.5, 48.0)	262	0.000*
**Total memory B cells T0**<br>**Total memory B cells T12**	32.3 (24.7, 41.1)<br>41.1 (37.6, 57.0)	16	0.000*
**Class switched memory B cells T0**<br>**Class switched memory B cells T12**	20.9 (17.7, 25.1)<br>26.9 (20.6, 31.0)	34	0.001*
**Class non switched memory B cells T0**<br>**Class non switched memory T12**	11.4 (5.5, 15.2)<br>14 (11.1, 26.2)	60	0.018

Wilcoxon signed-rank test for two related samples (before transplantation and one year after transplantation). Significance threshold: $p < 0.005$ (multiple testing correction)

.
